# Physicians’ attitudes and perceived diagnostic confidence in point-of-care ultrasound in gynecology and obstetrics (GO-POCUS): a prospective single-center implementation study with structured training

**DOI:** 10.1186/s12909-026-09799-z

**Published:** 2026-06-29

**Authors:** Sebastian Griewing, Florian Gerber, Zoe S. Oftring, Jonathan Bamberger, Alexandra Groß, Matthias Kalder, Malin Jansen, Corinna Keil, Johannes Knitza, Niklas Gremke, Uwe Wagner, Michael Leyer, Sebastian Kuhn

**Affiliations:** 1 Department of Medicine, Institute for Digital Medicine, Philipps-University of Marburg, Marburg, Germany; 2 Department of Business Administration, Research Group for Digitalization and Process Management, Philipps-University of Marburg, Marburg, Germany; 3 Department of Medicine, Clinic of Gynecology and Obstetrics, Philipps-University of Marburg, Marburg, Germany; 4 Department of Medicine, Philipps-University of Marburg, Clinic of Pediatrics, Marburg, Germany; 5 Commission Digital Medicine, German Society of Gynecology and Obstetrics, Berlin, Germany

**Keywords:** POCUS, Gynecology, Obstetrics, Implementation study, Technology acceptance

## Abstract

**Background:**

Point-of-care Ultrasound (POCUS) is increasingly introduced in obstetrics and gynecology as a focused bedside extension of conventional ultrasound, although evidence on its implementation in routine care remains limited. This study examined physicians’ attitudes towards POCUS, their perceived diagnostic confidence across different clinical scenarios, and their preference for POCUS compared with standard ultrasound devices. These outcomes were assessed during early implementation in routine care accompanied by structured training.

**Methods:**

In this prospective, longitudinal implementation study, 22 physicians from a university department of gynecology and obstetrics evaluated standard ultrasound devices at baseline (T0a), completed a structured hands-on POCUS training, and assessed POCUS immediately after training (T0b) and after 2 weeks (T1), 1 month (T2), and 3 months (T3) of clinical use. Evaluations were conducted using repeated quantitative surveys. Outcomes were attitude (4 items, 7-point Likert), perceived diagnostic confidence in obstetric and gynecologic scenarios (17 items, 7-point Likert), and device preference (7-point Likert and dichotomous). Quantitative analyses included descriptive statistics, paired tests, mixed-effects models, and non-parametric sensitivity analyses.

**Results:**

Attitude toward POCUS was significantly more favorable than attitude toward standard devices at baseline (T0a 3.69 vs. T0b 5.83; p < .001) and remained high throughout follow-up. Perceived diagnostic confidence for POCUS was not higher immediately after training but increased significantly over time in both obstetrics and gynecology after independent clinical use (both p < .001). Highest confidence was observed in focused bedside scenarios relevant to rapid orientation and immediate decision-making, including fetal vitality assessment, placental localization, amniotic fluid assessment, postvoid residual urine measurement, and urinary tract obstruction, whereas confidence remained lower for more complex applications such as cervical length assessment, Doppler-based examinations, and fetal growth restriction. Preference for POCUS was already high at baseline and remained stable over time.

**Conclusions:**

POCUS showed high acceptance in gynecologic and obstetric care from early implementation to routine use. Its clinical relevance appears greatest for focused mobile use and rapid bedside decision-making. These findings were observed during early implementation and support the role of POCUS as a complement to comprehensive ultrasound.

**Clinical trial registration:**

German Registry of Clinical Trials; registration number: DRKS 00036941; date of registration: July 16, 2025; title: GO-POCUS: Point-Of-Care UltraSound in Gynecology and Obstetrics: Attitude and Perceived Diagnostic Confidence among Physicians.

**Supplementary Information:**

The online version contains supplementary material available at 10.1186/s12909-026-09799-z.

## Background

Ultrasound is a first-line imaging modality in obstetrics and gynecology and has substantially shaped diagnostic practice in both fields since its clinical introduction in the 1960s [1–3]. Based on its non-invasive nature, broad availability, and real-time imaging capacity, it has become indispensable in routine care and emergency settings alike [2]. Recent advances, including portable devices, tele-ultrasound, elastography, and artificial intelligence, have further expanded its clinical utility [4–6].

Point-of-care Ultrasound (POCUS) represents a focused application of ultrasound performed directly at the bedside or point of care using mobile devices [7]. In contrast to comprehensive examinations on standard high-end systems, POCUS is intended to answer targeted clinical questions and support immediate clinical decision-making [7]. In obstetrics, POCUS has been reported to support confirmation of intrauterine pregnancy, assessment of fetal vitality, multiple pregnancy, and gestational age, as well as evaluation of placental location, cervical length, and amniotic fluid volume [4]. In gynecology, it has been shown to aid in the evaluation of abdominal pain, vaginal bleeding, abdominal distension, and pelvic masses [4]. Evidence from neonatology further highlights the growing relevance of focused ultrasound approaches across perinatal care settings [8].

POCUS has been reported to offer several potential advantages, including faster clinical assessment, improved workflow, and increased physician confidence in time-critical situations [7, 9–11]. However, the benefits of POCUS depend on adequate training and user competence [6, 12]. Limited experience may result in acquisition or interpretation errors with possible consequences for patient management. Accordingly, current recommendations emphasize structured training, certification, and continuous quality assurance [6, 12–14].

Successful implementation of POCUS therefore requires more than device availability. It depends on physician acceptance, perceived diagnostic value, integration into clinical workflows, and effective training strategies. Theoretical models of acceptance and use suggest that attitudes toward a technology and confidence in its utility are central determinants of adoption in practice [15, 16]. In obstetrics and gynecology, where POCUS is intended to complement rather than replace standard ultrasound systems, these factors are particularly relevant.

Despite increasing interest in POCUS in obstetrics and gynecology, real-world evidence on its implementation in clinical routine remains limited. Existing literature has mainly focused on potential indications, technical applications, and guideline recommendations, whereas physicians’ attitudes, perceived diagnostic confidence, and the effects of longitudinal training programs have received less attention in the literature [4]. Only a few studies have examined the practical implementation of POCUS in routine medical workflows, and these have focused predominantly on rural and low-resource settings, where POCUS has been considered particularly promising, without following structured teaching rationale [13, 17]. This is notable given that structured ultrasound curricula for medical students have shown beneficial effects on competence and integration of ultrasound into clinical reasoning in other settings [12, 14].

Against this background, this prospective observational study investigates physicians’ attitudes, their perceived diagnostic confidence across various gynecological and obstetric clinical scenarios, and their preference for POCUS versus standard ultrasound devices during its implementation in a high-resource, tertiary-care setting.

## Methods

### Study design

This prospective, longitudinal, observational implementation study was conducted at the Department of Gynecology and Obstetrics, University Hospital Marburg, Germany. The study investigated physicians’ attitudes towards point-of-care ultrasound (POCUS) and their perceived diagnostic confidence during the implementation of POCUS in routine clinical care, accompanied by structued training. The study was approved by the Ethics Committee of the Department of Medicine or Philipps-University Marburg on July 8, 2025 (25–79 BO) and registered in the German Clinical Trials Registry on July 16, 2025 (DRKS 00036941). Physicians in postgraduate training and board-certified specialists in the Department of Gynecology and Obstetrics were eligible to participate voluntarily. Following the provision of information about the study and written informed consent, data were collected in pseudonymized form via the Unipark platform (Tivian XI GmbH, Cologne, Germany) according to the requirements of the German General Data Protection Regulation (Datenschutz-Grundverordnung, DSGVO). All data were collected and stored in a pseudonymized form. Recruitment started on August 26, 2025 and lasted until January 28, 2026. This study is reported in accordance with the Strengthening the Reporting of Observational Studies in Epidemiology (STROBE) checklist for cohort studies (see Supplementary 1).

### Material

The handheld POCUS device used in the study was the Butterfly iQ3 (Butterfly Network, Inc., Burlington, Massachusetts, United States of America). Standard ultrasound devices routinely used in the department were the Samsung SonoAce R7 (Samsung Medison Co., Ltd., Seoul, Republic of Korea) in gynecology and the Fujifilm SonoSite M-Turbo in obstetrics (FUJIFILM SonoSite, Inc., Bothell, Washington, United States of America).

### Measurements and methods

The study comprised five measurement time points and followed a predefined implementation strategy. At baseline (T0), participants first evaluated the conventional ultrasound devices used in routine clinical care before the introduction of POCUS (T0a). This was followed by a structured 60-minute hands-on POCUS training session, which was mandatory for study participation and delivered by the principal investigator. To facilitate supervised practical training, participants were divided into two groups of almost equal size. The session followed a standardized training consisting of a 15-minute lecture on POCUS and the Butterfly iQ3 device, including an overview of the device’s functions, followed by a 10-minute live demonstration by the principal investigator addressing several relevant clinical scenarios. In addition, participants were introduced to the Butterfly educational library (Butterfly Academy), which provided access to on-demand educational content throughout the entire study. Participants then used the device themselves for 30 min under the supervision of the study investigators, with the opportunity to ask questions and receive individual instruction as needed. The training concluded with a 5-minute question-and-answer wrap-up. Immediately afterwards, participants completed the initial POCUS assessment (T0b).

**Fig. 1:**
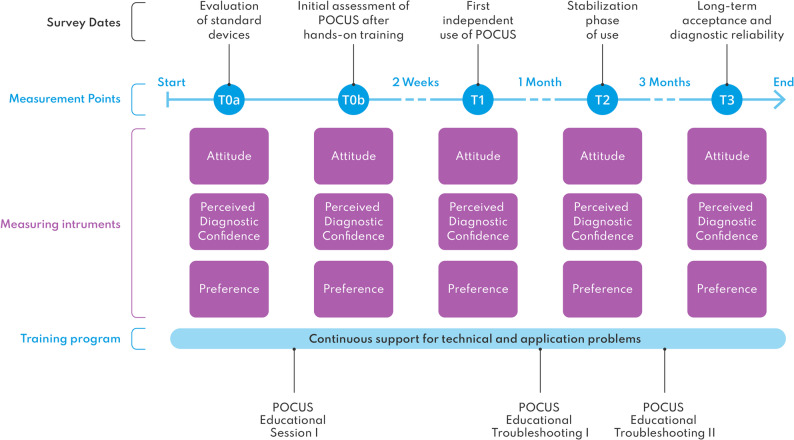
Overview of measurements and structured training

Follow-up measurements were conducted after 2 weeks (T1), 1 month (T2), and 3 months (T3) of independent POCUS use in routine clinical practice. Data collection was performed within a predefined time window of ± 5 days around each scheduled measurement point (see Fig. 1).

As part of the implementation strategy, participants were offered continuous technical support by the study investigators, enabling them to contact the study team throughout the study with technical or application-related questions. In addition, two optional troubleshooting sessions of approximately 30 min each were offered on a voluntary basis. Before these sessions, participants could submit technical difficulties or application-related questions to the principal investigator in advance. Alternatively, they could request additional hands-on training for specific clinical scenarios in which they wished to gain further experience. These sessions were intended to address individual support needs and provided an opportunity for further supervised hands-on practice, clarification of technical issues, and application training in relevant clinical scenarios.

The study assessed three outcomes: (1) attitude, (2) perceived diagnostic confidence, and (3) ultrasound device preference.

#### Attitude

Attitude toward the ultrasound device used at each measurement point was defined as the study’s primary outcome. Based on the affective component of Fishbein and Ajzen’s Reasoned Action Approach [15], this construct was measured using four items rated on a 7-point Likert scale (see Supplementary 2). Specifically, participants indicated the extent to which they perceived the use of the ultrasound device as advantageous, satisfying, important, and pleasant.

#### Perceived diagnostic confidence

Perceived diagnostic confidence was assessed as a secondary outcome using a 7-point Likert scale across 9 predefined obstetric and 8 gynecological clinical scenarios (see Supplementary 2). In obstetrics, these scenarios included fetal bradycardia, fetal biometry, fetal vitality assessment in the second and third trimesters, cervical length assessment, placental localization, amniotic fluid assessment, fetal growth abnormalities (small-/large-gestational age, SGA/LGA), umbilical artery Doppler, and fetal growth restriction (FGR). In gynecology, the assessed scenarios comprised first-trimester fetal vitality assessment, postvoid residual urine, urinary tract obstruction, focused assessment with sonography for abdominal bleeding (FAST), pleural effusion, ascites, postoperative seroma, and vascular visualization for venous access.

#### Ultrasound device preference

In addition, participants reported their preferred ultrasound device (POCUS vs. standard device) using both a 7-point Likert scale (1 for standard devices, 7 for POCUS) and a dichotomous measure (standard devices versus POCUS; see Supplementary 2).

### Statistical analyses

Initially, descriptive statistics were used to summarize participant characteristics and outcome measures. Continuous variables are reported as means and standard deviations (SD), and categorical variables as frequencies and percentages.

Baseline comparisons between the conventional ultrasound devices at T0a and POCUS immediately after initial training at T0b were performed using paired-samples t-tests. Wilcoxon signed-rank tests were additionally calculated as non-parametric sensitivity analyses. Paired effect sizes are reported as Cohen’s dz.

Longitudinal changes in POCUS-related outcomes were analyzed from T0b to T3 only, because T0a referred to standard ultrasound devices rather than POCUS use. These repeated-measures analyses were restricted to participants with complete data across all POCUS assessments. Linear mixed-effects models with random intercepts for participants and timepoint as a categorical fixed effect were used to evaluate overall time effects, complemented by Friedman tests as non-parametric repeated-measures analyses.

When appropriate, pairwise comparisons between timepoints were conducted using paired t-tests and Wilcoxon signed-rank tests with Bonferroni correction. For the dichotomous device-preference measure, changes across T0b–T3 were tested using Cochran’s Q test, with pairwise McNemar tests and Bonferroni-adjusted p-values where applicable.

Internal consistency was assessed using Cronbach’s alpha. For key paired contrasts, 95% confidence intervals for effect sizes were estimated using bootstrap resampling with 2,000 iterations. All tests were two-sided, and statistical significance was defined as *p* < .05.

All analyses were conducted in Python 3.14.3 using NumPy 2.4.3, pandas 3.0.1, SciPy 1.17.1, statsmodels 0.14.6, matplotlib 3.10.8, and seaborn 0.13.2.

## Results

### Participants

A total of 22 physicians started the study and completed both the baseline (T0a) and immediate post-training (T0b) assessments. One participant was lost to follow-up in T3. Thus, mean age was 36.57 years (SD = 8.29; range 27–60, *n* = 21 with complete demographic data), and mean professional experience was 9.48 years (SD = 7.97; range 1–33). Sex distribution was 13 female and 8 male participants. Regarding professional position, 11 (50.0%) participants were in postgraduate training, 6 (27.3%) were attending physicians, 2 (9.1%) were board-certified specialists, and 2 (9.1%) were senior attending physicians. Fourteen participants (63.6%) reported no subspecialty focus. Among those with a reported sub-specialty training, 2 participants (9.1%) indicated specialized perinatal care and obstetrics, 2 (9.1%) senology, 1 (4.5%) reproductive medicine, 1 (4.5%) perinatal care combined with gynecologic oncology, and 1 (4.5%) gynecologic oncology combined with senology (see Table 1).

**Table 1:** Participants´ demographics

Variable	Value
Age, years; mean (SD) [range]	36.57 (8.29) [27–60]; *n* = 21
Professional experience, years; mean (SD) [range]	9.48 (7.97) [1–33]; *n* = 21
Sex, n (%)	
Female	13 (59.1%)
Male	8 (36.4%)
Missing	1 (4.5%)
Training level, n (%)	
Postgraduate training	11 (50.0%)
Attending physician	6 (27.3%)
Board-certified specialist	2 (9.1%)
Senior attending physician	2 (9.1%)
Missing	1 (4.5%)
Subspecialty focus, n (%)	
None	14 (63.6%)
Perinatal care and obstetrics	2 (9.1%)
Senology	2 (9.1%)
Perinatal care and gynecologic oncology	1 (4.5%)
Gynecologic oncology and senology	1 (4.5%)
Reproductive medicine	1 (4.5%)
Missing	1 (4.5%)

At each follow-up assessment (T1, T2, T3), 21 of 22 responses were available. However, the set of responding participants differed across follow-up timepoints, such that longitudinal analyses requiring repeated observations across all POCUS assessments were restricted to the 19 participants with complete data from T0b through T3.

### Descriptive analyses

Table 2 summarizes the descriptive statistics for all outcome measures across the five assessment timepoints. At baseline, attitudes toward the standard ultrasound devices were lower than attitudes toward POCUS immediately after initial training, whereas diagnostic confidence for the standard devices was initially similar to or slightly higher than confidence for POCUS. Across follow-up, attitude toward POCUS remained high, and both obstetric and gynecologic diagnostic confidence showed higher mean values than at the immediate post-training assessment. Device preference also remained on the POCUS-favoring side of the scale throughout follow-up. Cronbach’s alpha ranged from 0.751 to 0.960 for the attitude scale, from 0.921 to 0.951 for obstetric diagnostic confidence, and from 0.877 to 0.964 for gynecologic diagnostic confidence (see Supplementary 3).

**Table 2:** Descriptive analyses

Outcomes	Point of Measurement	*N*	Mean	SD	Median	Q1	Q3	Min	Max
Attitude	**T0a**	22	3.69	1.16	3.50	2.81	4.44	1.75	6.25
	**T0b**	22	5.83	0.64	5.75	5.50	6.00	4.75	7.00
	**T1**	19	5.88	0.84	6.00	5.38	6.50	4.00	7.00
	**T2**	19	5.79	1.23	6.00	5.12	6.75	2.75	7.00
	**T3**	19	6.16	0.94	6.25	5.75	7.00	4.00	7.00
Perceived Diagnostic Confidence: *Obstetrics*	**T0a**	22	4.03	1.47	3.56	3.03	4.94	1.00	7.00
	**T0b**	22	3.85	1.17	3.94	3.31	4.61	1.00	6.11
	**T1**	19	4.92	1.18	4.56	4.00	5.94	3.44	7.00
	**T2**	19	4.95	1.08	4.78	4.06	5.94	3.11	6.56
	**T3**	19	5.11	0.97	5.11	4.56	5.78	3.22	6.67
Perceived Diagnostic Confidence: *Gynecology*	**T0a**	22	4.89	0.96	4.88	4.06	5.34	3.75	6.75
	**T0b**	22	4.57	1.11	4.62	4.38	5.00	2.00	7.00
	**T1**	19	5.57	1.03	5.62	4.88	6.38	3.88	7.00
	**T2**	19	5.52	1.21	5.62	4.44	6.44	3.25	7.00
	**T3**	19	5.84	0.96	5.75	5.50	6.75	4.00	7.00
Preference	**T0a**	22	4.64	1.05	4.50	4.00	5.00	2.00	7.00
	**T0b**	22	4.82	1.01	5.00	4.00	5.00	3.00	7.00
	**T1**	19	5.21	1.58	5.00	4.50	6.50	1.00	7.00
	**T2**	19	5.32	1.45	6.00	4.00	6.00	2.00	7.00
	**T3**	19	5.11	1.49	5.00	4.00	6.00	2.00	7.00

T0a and T0b descriptives are based on the full sample (N = 22), whereas T1-T3 descriptives are based on the 19 complete cases included in the longitudinal analyses. All scales were scored on Likert scale from 1 to 7. Values represent averaged scale scores across items; therefore, minima (Min) and maxima (Max) may be non-integer values. T0a = baseline assessment of standard ultrasound devices before POCUS introduction. T0b = immediate post-training POCUS assessment. T1 = 2-week follow-up. T2 = 1-month follow-up. T3 = 3-month follow-up. SD = standard deviation, Q1 = lower quartile (25th percentile), Q3 = upper quartile (75th percentile)

### Attitude

**Fig. 2:**
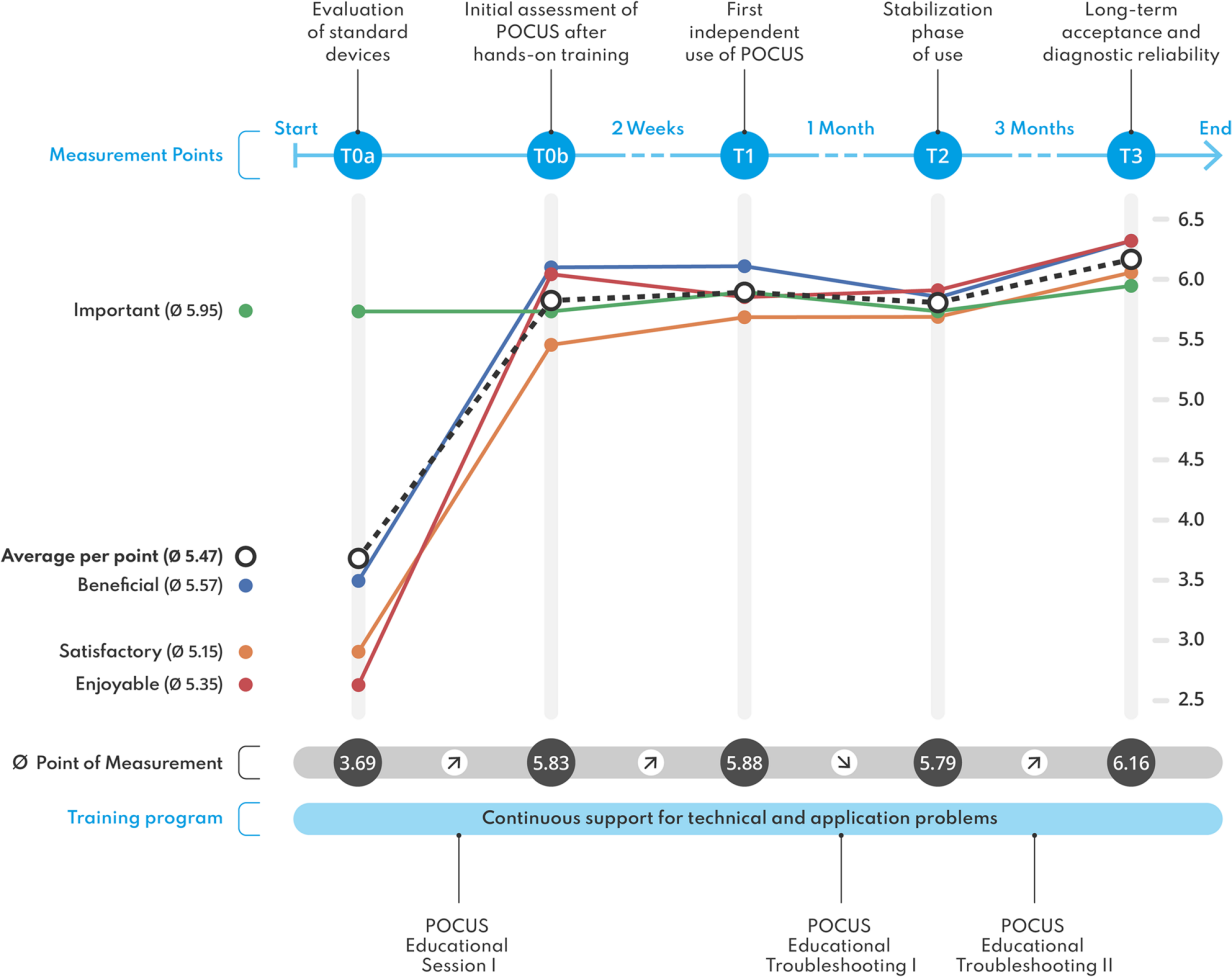
Longitudinal development of attitude

Ratings of attitude were significantly higher for POCUS at the first post-training assessment (T0b) than for the standard devices at baseline (T0a). In the full sample (*N* = 22), the mean overall attitude score was 3.69 (SD = 1.16) for the standard devices at T0a and 5.83 (SD = 0.64) for POCUS at T0b, corresponding to a very large effect (paired t-test: *p* < .001; Wilcoxon signed-rank test: *p* < .001; dz = 2.03). The largest differences between the baseline assessment of the standard devices and the initial assessment of POCUS were observed for the items beneficial (3.50 vs. 6.09), satisfactory (2.91 vs. 5.45), and enjoyable (2.64 vs. 6.05), whereas importance was already rated comparatively high for the standard devices at baseline (5.73). Across the longitudinal follow-up in the complete-case sample (*N* = 19), attitude toward POCUS remained high (T0b: 5.92; T1: 5.88; T2: 5.79; T3: 6.16), with no evidence of a significant overall change over time (mixed-effects model: *p* = .206; Friedman test: *p* = .110) (see Table 3; Fig. 2).

**Table 3:** Results for attitude

Item	Standard devices	POCUS			
	T0a (*N* = 22)	T0b (*N* = 22)	T1 (*N* = 19)	T2 (*N* = 19)	T3 (*N* = 19)
Beneficial	3.50 (1.85)	6.09 (0.87)	6.11 (0.88)	5.84 (1.42)	6.32 (0.95)
Satisfactory	2.91 (1.34)	5.45 (0.86)	5.68 (1.11)	5.68 (1.25)	6.05 (0.97)
Important	5.73 (1.32)	5.73 (0.98)	5.89 (0.99)	5.74 (1.19)	5.95 (1.27)
Enjoyable	2.64 (1.47)	6.05 (0.65)	5.84 (1.01)	5.89 (1.33)	6.32 (0.89)
Average Score	3.69 (1.16)	5.83 (0.64)	5.88 (0.84)	5.79 (1.23)	6.16 (0.94)

Values given in the following format: mean average (standard deviation). T0a and T0b descriptives are based on the full sample (*N* = 22), whereas T1-T3 descriptives are based on the 19 complete cases included in the longitudinal analyses. All scales were scored on Likert scale from 1 to 7. T0a = baseline assessment of standard ultrasound devices before POCUS introduction. T0b = immediate post-training POCUS assessment. T1 = 2-week follow-up. T2 = 1-month follow-up. T3 = 3-month follow-up

### Perceived diagnostic confidence

**Fig. 3:**
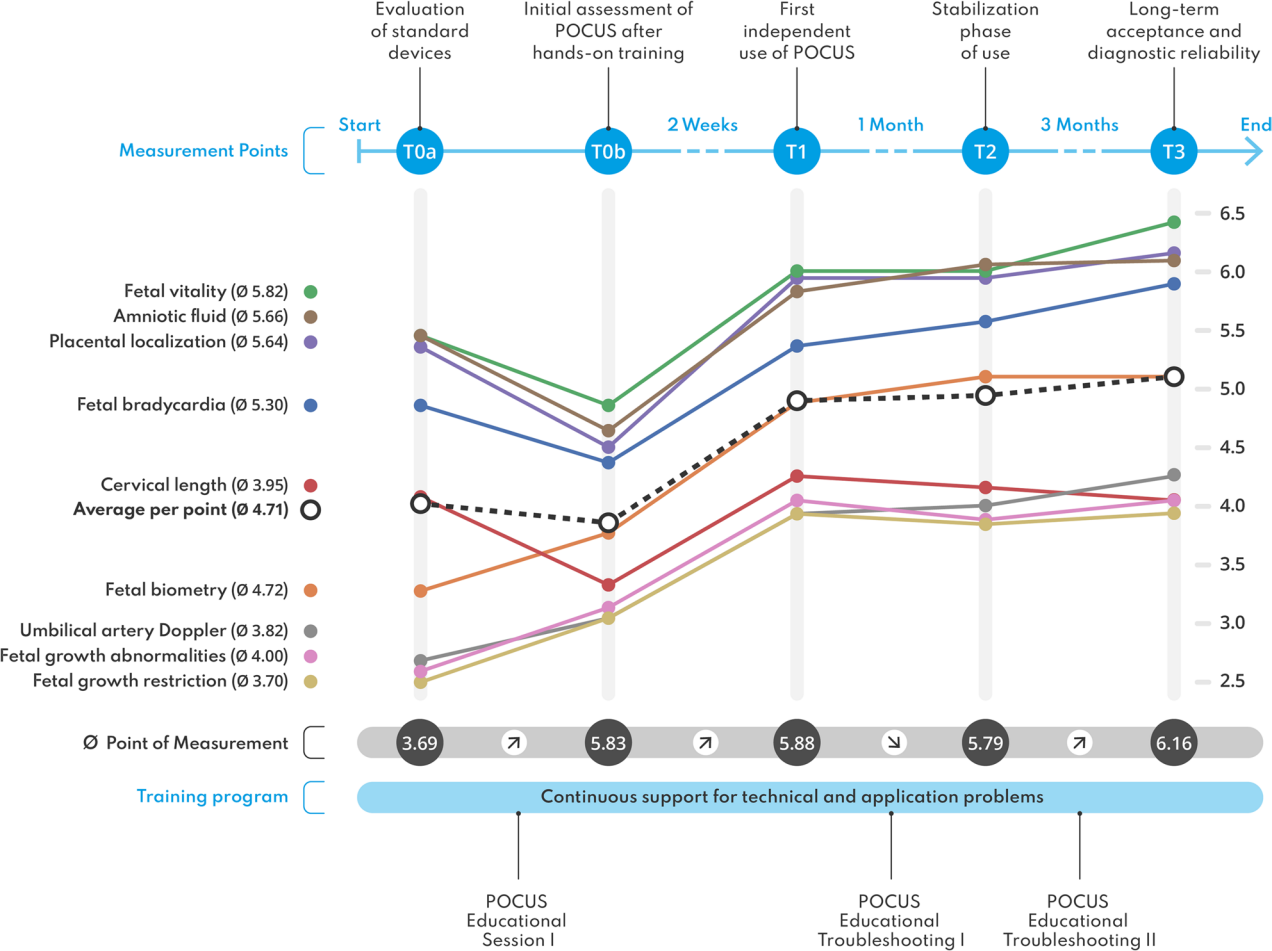
Longitudinal development of perceived diagnostic confidence in obstetrics

Compared with baseline ratings for the standard devices (T0a, mean 4.03), the first assessment of POCUS immediately after initial training (T0b, mean 3.85) showed slightly lower overall diagnostic confidence. This difference was not statistically significant (t(21) = -0.55, *p* = .591, dz = -0.12). However, confidence increased after the first independent clinical use (T1, mean 4.92) and remained stable through T2 (4.95) and T3 (5.11). In the complete-case sample (*N* = 19), the longitudinal effect was significant in both the mixed-effects model (chi2(3) = 24.98, *p* < .001) and the Friedman test (chi2(3) = 17.54, *p* < .001), and the change from T0b to T3 corresponded to a large effect size (dz = 1.02, 95% CI [0.66, 1.62]). Bonferroni-corrected pairwise comparisons showed significant increases from T0b to T1 (+ 0.85, corrected *p* = .014), T0b to T2 (+ 0.89, corrected *p* = .027), and T0b to T3 (+ 1.05, corrected *p* = .002). Across individual obstetric applications, the highest average confidence ratings during follow-up were observed for fetal vitality in the second and third trimester (T1-T3 range: 6.00-6.42), placental localization (5.95–6.16), amniotic fluid assessment (5.84–6.11), and fetal bradycardia (5.37–5.89), whereas confidence remained lower for FGR (3.84–3.95), fetal growth abnormalities (SGA and LGA) (3.76–4.05), umbilical artery Doppler (3.95–4.26), and cervical length (4.05–4.26) (see Table 4; Fig. 3). Because item-level inferential testing was limited by the small sample size, clinical heterogeneity within the obstetric confidence composite was addressed in an exploratory cluster-based analysis. Focused bedside and rapid-assessment scenarios showed consistently higher confidence ratings than advanced obstetric assessment scenarios, while both clusters increased significantly from T0b to T3 (see Supplementary 4).

**Table 4:** Results for perceived diagnostic confidence in obstetrics

Clinical scenario	T0a (*N* = 22)	T0b (*N* = 22)	T1 (*N* = 19)	T2 (*N* = 19)	T3 (*N* = 19)	Average T0b-T3
Fetal bradycardia	4.86 (1.88)	4.36 (1.62)	5.37 (1.26)	5.58 (1.17)	5.89 (0.94)	5.30
Fetal biometry	3.27 (1.96)	3.77 (1.23)	4.89 (1.33)	5.11 (1.15)	5.11 (1.29)	4.72
Fetal vitality (2nd/3rd trimester)	5.45 (1.53)	4.86 (1.64)	6.00 (1.25)	6.00 (1.11)	6.42 (0.96)	5.82
Cervical length	4.09 (2.14)	3.32 (1.36)	4.26 (1.88)	4.16 (1.68)	4.05 (1.58)	3.95
Placental localization	5.36 (1.43)	4.50 (1.41)	5.95 (1.08)	5.95 (1.18)	6.16 (1.07)	5.64
Amniotic fluid	5.45 (1.34)	4.64 (1.53)	5.84 (1.01)	6.05 (1.08)	6.11 (1.05)	5.66
Fetal growth abnormalities (SGA/LGA)	2.59 (1.79)	3.14 (1.21)	4.05 (1.78)	3.89 (1.70)	4.05 (1.61)	4.00
Umbilical artery Doppler	2.68 (1.89)	3.05 (1.21)	3.95 (1.75)	4.00 (1.49)	4.26 (1.59)	3.82
Fetal growth restriction (FGR)	2.50 (1.68)	3.05 (1.36)	3.95 (1.75)	3.84 (1.61)	3.95 (1.61)	3.70
Average Score	4.03 (1.47)	3.85 (1.17)	4.92 (1.18)	4.95 (1.08)	5.11 (0.97)	4.71

Values given in the following format: mean average (standard deviation). T0a and T0b descriptives are based on the full sample (N = 22), whereas T1-T3 descriptives are based on the 19 complete cases included in the longitudinal analyses. All scales were scored on Likert scale from 1 to 7. T0a = baseline assessment of standard ultrasound devices before POCUS introduction. T0b = immediate post-training POCUS assessment. T1 = 2-week follow-up. T2 = 1-month follow-up. T3 = 3-month follow-up

**Fig. 4:**
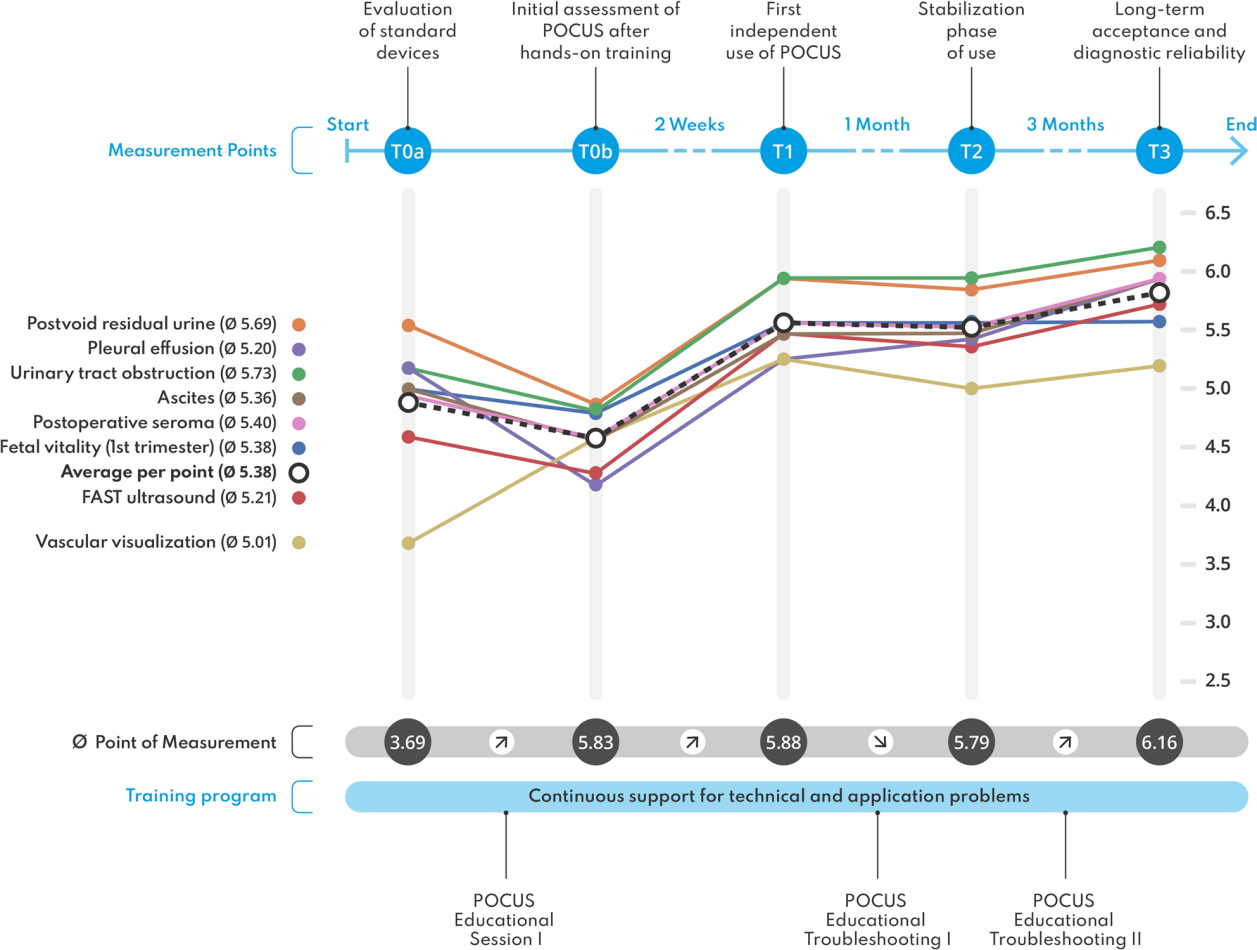
Longitudinal development of perceived diagnostic confidence in gynecology

Similarly, the first post-training assessment of POCUS (T0b, mean 4.57) showed slightly lower overall diagnostic confidence than baseline ratings for the standard devices (T0a, mean 4.89). This difference was likewise not significant (t(21) = -1.17, *p* = .257, dz = -0.25). Confidence increased substantially after the first independent clinical use (T1, mean 5.57) and remained high through T2 (5.52) and T3 (5.84). In the complete-case sample, the longitudinal effect was significant in the mixed-effects model (chi2(3) = 24.28, *p* < .001) and in the Friedman test (chi2(3) = 12.25, *p* = .007), with a large T0b-to-T3 effect size (dz = 0.90, 95% CI [0.51, 1.49]). Pairwise comparisons showed significant increases from T0b to T1 (+ 0.91, corrected *p* = .015) and from T0b to T3 (+ 1.18, corrected *p* = .006), whereas the T0b-to-T2 comparison (+ 0.86) was significant before correction but not after Bonferroni adjustment (corrected *p* = .077). During follow-up, the highest confidence ratings were observed for postvoid residual urine measurement (5.84–6.11) and assessment of urinary tract obstruction (5.95–6.21), whereas confidence remained comparatively lower for vascular visualization (5.00–5.26), FAST ultrasound (5.37–5.74), and pleural effusion assessment (5.26–5.95) (see Fig. 4; Table 5).

**Table 5:** Results for perceived diagnostic confidence in gynecology

Clinical scenario	T0a (*N* = 22)	T0b (*N* = 22)	T1 (*N* = 19)	T2 (*N* = 19)	T3 (*N* = 19)	Average T0b-T3
Fetal vitality (1st trimester)	5.00 (1.31)	4.77 (1.27)	5.58 (0.96)	5.58 (1.17)	5.58 (1.30)	5.38
Postvoid residual urine	5.55 (0.86)	4.86 (1.39)	5.95 (0.97)	5.84 (1.07)	6.11 (0.81)	5.69
Urinary tract obstruction	5.18 (1.18)	4.82 (1.33)	5.95 (0.97)	5.95 (1.22)	6.21 (0.85)	5.73
FAST ultrasound	4.59 (1.62)	4.27 (1.24)	5.47 (1.39)	5.37 (1.54)	5.74 (1.33)	5.21
Pleural effusion	5.18 (1.05)	4.18 (1.33)	5.26 (1.48)	5.42 (1.39)	5.95 (1.18)	5.20
Ascites	5.00 (1.38)	4.55 (1.30)	5.47 (1.47)	5.47 (1.50)	5.95 (1.31)	5.36
Postoperative seroma	4.95 (1.21)	4.55 (1.26)	5.58 (1.35)	5.53 (1.35)	5.95 (1.22)	5.40
Vascular visualization	3.68 (1.64)	4.55 (1.18)	5.26 (0.99)	5.00 (1.49)	5.21 (1.13)	5.01
Average Score	4.89 (0.96)	4.57 (1.11)	5.57 (1.03)	5.52 (1.21)	5.84 (0.96)	5.38

Values given in the following format: mean average (standard deviation). T0a and T0b descriptives are based on the full sample (*N* = 22), whereas T1-T3 descriptives are based on the 19 complete cases included in the longitudinal analyses. All scales were scored on Likert scale from 1 to 7. T0a = baseline assessment of standard ultrasound devices before POCUS introduction. T0b = immediate post-training POCUS assessment. T1 = 2-week follow-up. T2 = 1-month follow-up. T3 = 3-month follow-up

Overall, diagnostic confidence did not improve immediately after training alone but increased after subsequent clinical POCUS use. Individual-level change patterns further showed that improvement was broadly distributed across participants, with 63.2% improving in obstetric confidence and 68.4% improving in gynecologic confidence between T0b and T3.

Further results of the inferential analyses, including post hoc pairwise comparisons, are presented in Supplementary 5.

### Ultrasound device preference

At baseline (T0a), the mean Likert-scale preference rating for POCUS compared with standard devices was 4.64, increasing to 4.82 after the initial hands-on POCUS introduction (T0b). However, this immediate change was not statistically significant (paired t-test: *p* = .463; Wilcoxon signed-rank test: *p* = .521; dz = 0.16). In the dichotomous measure, 72.7% of physicians (16/22) preferred POCUS over standard devices at T0a, despite not having used POCUS previously, this proportion increased to 81.8% (18/22) after training.

At the first independent use of POCUS after two weeks (T1), the mean Likert-scale preference rating for POCUS compared with standard devices increased to 5.21. In the complete-case sample (*N* = 19), these longitudinal changes from T0b onward were not statistically significant (mixed-effects model: chi2(3) = 1.75, *p* = .626; Friedman test: chi2(3) = 2.79, *p* = .426; dz T0b to T3 = 0.13, 95% CI [-0.33, 0.68]). Pairwise comparisons likewise showed no significant change over time (all Bonferroni-corrected p values = 1.000). In the dichotomous measure, POCUS remained the preferred device at all follow-up timepoints, with 89.5% of physicians (17/19) preferring POCUS at T1, 89.5% (17/19) at T2, and 84.2% (16/19) at T3. The corresponding Cochran’s Q test for T0b-T3 indicated no significant change in binary preference over time (Q = 0.53, *p* = .912) (see Table 6).

**Table 6:** Results for device preference

Outcome Measure	Timepoint	*N*	Result	Test Information
Device Preference (Likert)	T0a	22	4.64 (1.05)	Baseline standard-device comparison; T0a vs. T0b: paired t-test *p* = .463; dz = 0.16
	T0b	22	4.82 (1.01)	Mixed-effects model: *p* = .626; Friedman: *p* = .426
	T1	19	5.21 (1.58)	
	T2	19	5.32 1.45)	
	T3	19	5.11 (1.49)	
Device Preference for POCUS (Dichotomous)	T0a	22	16 (72.7%)	Baseline standard-device comparison
	T0b	22	18 (81.8%)	Cochran’s Q (T0b-T3) = 0.53, *p* = .912
	T1	19	17 (89.5%)	
	T2	19	17 (89.5%)	
	T3	19	16 (84.2%)	

Values given in the following format: mean average (standard deviation) for Device Preference (Likert), scored on Likert scale from 1 to 7. Absolute number of participants preferring POCUS (percentage % of total number of participants) for Device Preference, evaluated in dichotomous manner

## Discussion

Previous studies have highlighted the potential value of POCUS across a range of clinical applications in obstetrics and gynecology [4, 10, 18]. Nevertheless, these studies lack real-world implementation data or are limited to low-resource and rural care settings [13, 17, 19]. Against this background, the present study was undertaken to examine how POCUS is perceived during its early implementation in routine gynecologic and obstetric care at a high-resource tertiary care provider, a context in which previous research has provided only limited evidence on physicians’ attitudes, perceived diagnostic confidence, and their longitudinal development.

### Principal findings

The central findings of this study are that physicians’ attitudes toward POCUS in obstetrics and gynecology were consistently positive during early implementation at a tertiary care center. This is particularly noteworthy because previous implementation-related research was limited to the examination of ultrasound training in medical students, whereas the present study transfers these insights to routine gynecologic and obstetric care [6, 12, 14]. Attitude toward POCUS was already high immediately after initial training, was significantly more favorable than attitudes toward standard ultrasound devices at baseline, and remained high throughout follow-up, indicating sustained acceptance during the early implementation phase. Although ultrasound was considered important irrespective of device type, the advantages of POCUS were seen in its ratings as beneficial, satisfactory, and enjoyable. These findings suggest that the successful adoption of POCUS is consistent with Fishbein and Ajzen’s Reasoned Action Approach, in that acceptance depends not only on device availability but also on whether clinicians perceive the technology as beneficial, satisfactory, important and enjoyable for their routine practice [15].

### Further findings

Beyond attitude, perceived diagnostic confidence is of particular clinical relevance since implementing POCUS in gynecology and obstetrics requires not only general acceptance of the technology but also confidence in its appropriate use across specific clinical scenarios. To our knowledge, this has not previously been examined in a prospective real-world study of early POCUS implementation in routine gynecologic and obstetric care. This is especially important in the specialty context of gynecology and obstetrics, where handheld ultrasound must be applied in a focused manner, with clear awareness of its diagnostic boundaries and appropriate differentiation from comprehensive ultrasound. The present study therefore adds important insight by demonstrating both indication-specific differences in perceived diagnostic confidence and its longitudinal development during early implementation.

Thus, the development of perceived diagnostic confidence differed from the development of attitude. Confidence did not improve immediately after initial training but increased after the first period of independent clinical use and remained higher thereafter. However, the immediate post-training assessment should be interpreted with caution. Because the initial 60-minute training session was brief and could not provide hands-on experience across all 17 heterogeneous clinical scenarios, T0b ratings may partly reflect anticipated rather than fully experience-based confidence. Accordingly, the subsequent increase in confidence should be interpreted as an observed development during early clinical implementation and repeated clinical exposure, not as evidence of an immediate training effect alone. Furthermore, the study did not collect participant-level data on the frequency of POCUS device use. Therefore, the extent to which frequency of exposure influenced perceived diagnostic confidence cannot be determined within the current study design. These limitations warrant further evaluation of the educational effects of POCUS in gynecology and obstetrics using more extensive study designs, such as case-control studies or randomized controlled trials.

Current reviews and guidelines emphasize that POCUS is intended to answer focused bedside questions and requires structured training, awareness of limitations, and appropriate escalation to comprehensive ultrasound where needed [10, 11, 20, 21]. Immediate training may therefore promote general interest and acceptance while simultaneously making users more aware of the boundaries of their own competence to this point in time, whereas only repeated clinical application appears to consolidate confidence. This interpretation also aligns with broader ultrasound education literature showing that training during implementation supports the integration of ultrasound into clinical reasoning, while skill development depends on practice beyond initial teaching sessions [6, 12, 22]. In the present single-arm implementation study, however, the independent contribution of training cannot be separated from the effects of clinical exposure, local implementation conditions, and repeated use during follow-up. Furthermore, the extent of clinical exposure to POCUS was not recorded as part of the study design and therefore, causal interpretations regarding the effect of exposure frequency cannot be inferred. Nevertheless, in the present study, the highest confidence ratings were observed in clinical scenarios that mainly serve rapid bedside orientation and immediate decision-making, including fetal vitality assessment, placental localization, amniotic fluid assessment, postvoid residual urine measurement, and urinary tract obstruction. By contrast, confidence remained lower in more complex applications such as cervical length assessment, Doppler-based examinations, and growth restriction. The exploratory cluster-based analysis further supports this interpretation by showing consistently higher confidence ratings for focused bedside and rapid-assessment scenarios than for advanced obstetric assessment scenarios. Thus, the overall increase in perceived diagnostic confidence should not be interpreted as uniform across all potential POCUS applications. Rather, it appears to reflect particularly strong perceived applicability for focused bedside tasks, while more advanced obstetric assessments may require more extensive training, repeated supervised practice, or continued use of comprehensive ultrasound systems [4]. This distinction is clinically important, because the mere technical feasibility of performing a given examination with a handheld device does not necessarily mean that its use is equally meaningful or appropriate within routine care processes. In this sense, POCUS in obstetrics and gynecology appears to be perceived less as a tool for detailed diagnostic work-up and more as a focused instrument for rapid clinical orientation in selected bedside scenarios.

While perceived diagnostic confidence primarily reflects whether physicians feel able to use POCUS appropriately in specific clinical scenarios, device preference captures a broader implementation-related judgment regarding the perceived role of handheld ultrasound in relation to established standard devices within routine gynecologic and obstetric care. Preference for POCUS was already high at baseline and remained stable over time without significant change. This indicates that handheld ultrasound was perceived favorably from the outset and maintained this position throughout early routine use.

### Limitations

Although this study is, to our knowledge, the first prospective real-world implementation study of handheld POCUS in routine obstetric and gynecologic care in a high-resource tertiary care setting, several limitations should be considered when interpreting the findings.

First, the study assessed perceived diagnostic confidence rather than actual diagnostic performance. While confidence is a highly relevant implementation-related outcome, it cannot be equated with diagnostic accuracy. Future studies should therefore complement self-reported outcomes with objective, formalized assessments of image acquisition and interpretation.

Second, time-related outcomes were not measured. This is relevant because improved workflow is often assumed to be an advantage of handheld POCUS, yet previous research has treated time efficiency as an important empirical question rather than a given benefit. Future studies in gynecology and obstetrics should therefore directly assess examination time, workflow integration, and whether handheld POCUS provides measurable process advantages in routine care.

Third, standard ultrasound devices were not reassessed at T1-T3, so comparisons between later POCUS assessments and baseline ratings for standard devices at T0a should be interpreted with caution. Improvements observed over time may not exclusively reflect POCUS-related effects, but may also be influenced by broader learning effects, growing general ultrasound experience, or increasing familiarity with the assessed clinical scenarios.

Fourth, the study was conducted in a single tertiary care department with a relatively small sample size. This limits generalizability and raises the possibility that the observed implementation effects were partly shaped by favorable local conditions, departmental culture, and organizational readiness for innovation. Larger multicenter studies and, where feasible, randomized or controlled comparative designs are warranted to confirm the generalizability of these findings and to evaluate the independent contribution of structured educational programs to POCUS implementation more robustly.

Fifth, potential sources of bias should be considered when interpreting the findings. The principal investigator was involved in several stages of the study, including study design, training delivery, participant support and data analysis. Although this close involvement may have supported consistent study implementation, it may also have introduced investigator bias, particularly in the relation to participant engagement, perceived expectations and interpretation of study outcomes. In addition, repeated POCUS-specific assessments and participants´ awareness of being observed during an implementation study may have contributed to Hawthorne effects, whereby participants modified their behavior or engagement because they knew they were being studied. Participants may also have inferred favorable expectations torward POCUS from the study context, the structured training program, investigator support and repeated assessments. These mechanisms may have influenced self-reported attitudes, perceived diagnostic confidence and reported preference of POCUS. Therefore, the magnitude of the observed changes should be interpreted with caution, particularly for outcomes based on subjective self-report.

Sixth, POCUS use frequency and intensity were not systematically recorded during follow-up. In the dynamic clinical environment, particularly during obstetric emergency situations, reliable logging of each POCUS examination was not feasible. Therefore, changes in perceived diagnostic confidence cannot be directly linked to the amount, frequency, or type of individual POCUS exposure during the follow-up period. The absence of exposure-frequency data also limits conclusions regarding possible relationships between clinical POCUS use and changes in confidence, competence or adoption. Future implementation studies should consider feasible approaches for capturing POCUS exposure during routine care, such as simplified logbooks, device-based usage data or periodic structured self-report, while minimizing additional documentation burden.

### Future research

Future studies should complement self-reported implementation outcomes with objective performance measures and directly evaluate workflow-related outcomes including examination time and process integration. Repeated follow-up assessment of standard ultrasound devices would also help to distinguish POCUS-specific effects from more general gains in ultrasound experience. In addition, semi-structured interviews based on the Extended Unified Theory of Acceptance and Use of Technology (UTAUT II) framework by Venkatesh et al. were conducted with participants as part of the GO-POCUS (DRKS 00036941) study and are currently being analyzed [16]. Although the sample size was limited, this qualitative component is expected to yield further detailed insight into the mechanisms underlying early POCUS adoption in routine gynecologic and obstetric care. Larger multicenter studies and, where feasible, randomized controlled trials are warranted to confirm the generalizability of these findings and to evaluate the effect of structured educational programs on POCUS implementation more robustly.

## Conclusions

In conclusion, handheld POCUS demonstrated high overall acceptance in routine gynecologic and obstetric care. Its clinical value appears to be particularly relevant for focused mobile use, rapid bedside orientation, and immediate decision-making rather than for detailed diagnostic work-up. These findings further underline that POCUS represents a relevant addition to imaging in obstetrics and gynecology, complementing comprehensive ultrasound rather than replacing it. The observed findings occurred during early routine clinical implementation accompanied by structured training.

## Supplementary information

Supplementary Material 1.

Supplementary Material 2.

Supplementary Material 3.

Supplementary Material 4.

Supplementary Material 5.

## Data Availability

Data are provided within the manuscript or supplementary material. Further datasets generated and analyzed during the current study are available from the corresponding author on reasonable request.

## Abbreviations

AC: Abdominal circumference
AFI: Amniotic fluid index
BPD: Biparietal diameter
DRKS: Deutsches Register Klinischer Studien
DSGVO: Datenschutz–Grundverordnung
FAST: Focused assessment with sonography for abdominal bleeding
FGR: Fetal growth restriction
FL: Femur length
GO: POCUS–Point–of–Care Ultrasound in Gynecology and Obstetrics
HC: Head circumference
LGA: Large for gestational age
POCUS: Point–of–care ultrasound
SD: Standard deviation
SDP: Single deepest pocket
SGA: Small for gestational age
T0a: Baseline assessment of standard ultrasound devices before POCUS introduction
T0b: Immediate post–training POCUS assessment
T1: 2–week follow–up
T2: 1–month follow–up
T3: 3–month follow–up
