# Supplementary material for: Physicians’ attitudes and perceived diagnostic confidence in point-of-care ultrasound in gynecology and obstetrics (GO-POCUS): a prospective single-center implementation study with structured training
Source: BMC Med Educ. 2026 Jun 29;26:1043. doi: 10.1186/s12909-026-09799-z (PMC13321536; doi:10.1186/s12909-026-09799-z)
Supplement: Supplementary file 1 — Supplementary Material 1. [file 12909_2026_9799_MOESM1_ESM.docx]

**Supplementary 2**

**Attitude** (7-point Likert each)

I find the use of POCUS…

(1)… beneficial.

(2)… satisfactory.

(3)… important.

(4)… enjoyable.

**Perceived Diagnostic Confidence** (7-point Likert each)

***Obstetrics:***

1. How confident do you feel when diagnosing fetal bradycardia using POCUS?

2. How confident do you feel when performing fetal biometry to determine biometric parameters (e.g., BPD, HC, AC, and FL) of the fetus using POCUS?

3. How confident are you in monitoring fetal vitality in the 2nd and 3rd trimesters (e.g., fetal heart rate, fetal movement) using POCUS?

4. How confident are you in determining cervical length using POCUS?

5. How confident do you feel when assessing placental location using POCUS?

6. How confident do you feel when determining amniotic fluid volume using POCUS (e.g., AFI, SDP)?

7. How confident do you feel when assessing fetal growth abnormalities (Small for Gestational Age [SGA] and Large for Gestational Age [LGA]) using POCUS?

8. How confident do you feel when assessing fetal Doppler parameters of the umbilical artery using POCUS?

9. How confident do you feel when assessing fetal growth restriction (FGR) using POCUS?

***Gynecology:***

1. How confident do you feel using POCUS to assess fetal vitality in the first trimester (e.g., heartbeat, movement, intra-amniotic fluid)?

2. How confident do you feel using POCUS to determine residual urine volume?

3. How confident are you in ruling out a urinary tract obstruction (assessment of the degree of renal congestion) using POCUS?

4. How confident are you in using POCUS for FAST ultrasound to rule out bleeding?

5. How confident are you in detecting or ruling out a pleural effusion using POCUS?

6. How confident are you in assessing ascites using POCUS?

7. How confident do you feel when detecting a postoperative seroma (e.g., after transverse laparotomy or breast surgery) using POCUS?

8. How confident do you feel when visualizing vessels (e.g., for placing an indwelling venous catheter) using POCUS?

**Device preference**

1. Do you tend to use the standard device or POCUS? (7-point Likert)
2. Please choose which device you'd like to use: (Dichotomous)
