# Supplementary material for: Physicians’ attitudes and perceived diagnostic confidence in point-of-care ultrasound in gynecology and obstetrics (GO-POCUS): a prospective single-center implementation study with structured training
Source: BMC Med Educ. 2026 Jun 29;26:1043. doi: 10.1186/s12909-026-09799-z (PMC13321536; doi:10.1186/s12909-026-09799-z)
Supplement: Supplementary file 2 — Supplementary Material 2. [file 12909_2026_9799_MOESM2_ESM.docx]

**Supplementary 3:**

**Internal consistency of composite scales across timepoints**

| **Scale** | **Timepoint** | **Number of items** | **Cronbach’s α** |
| --- | --- | --- | --- |
| Attitude | T0a | 4 | .775 |
| Attitude | T0b | 4 | .751 |
| Attitude | T1 | 4 | .870 |
| Attitude | T2 | 4 | .960 |
| Attitude | T3 | 4 | .893 |
| Diagnostic confidence: Obstetrics | T0a | 9 | .946 |
| Diagnostic confidence: Obstetrics | T0b | 9 | .943 |
| Diagnostic confidence: Obstetrics | T1 | 9 | .921 |
| Diagnostic confidence: Obstetrics | T2 | 9 | .951 |
| Diagnostic confidence: Obstetrics | T3 | 9 | .932 |
| Diagnostic confidence: Gynecology | T0a | 8 | .877 |
| Diagnostic confidence: Gynecology | T0b | 8 | .949 |
| Diagnostic confidence: Gynecology | T1 | 8 | .942 |
| Diagnostic confidence: Gynecology | T2 | 8 | .964 |
| Diagnostic confidence: Gynecology | T3 | 8 | .924 |

**Note.** Cronbach’s α was calculated separately for each composite scale at each timepoint. The attitude scale comprised four items, obstetric diagnostic confidence comprised nine clinical scenarios, and gynecologic diagnostic confidence comprised eight clinical scenarios. Values indicate acceptable to excellent internal consistency across all timepoints. T0a = baseline assessment of standard ultrasound devices before POCUS introduction; T0b = immediate post-training POCUS assessment; T1 = 2-week follow-up; T2 = 1-month follow-up; T3 = 3-month follow-up.
