# Supplementary material for: Physicians’ attitudes and perceived diagnostic confidence in point-of-care ultrasound in gynecology and obstetrics (GO-POCUS): a prospective single-center implementation study with structured training
Source: BMC Med Educ. 2026 Jun 29;26:1043. doi: 10.1186/s12909-026-09799-z (PMC13321536; doi:10.1186/s12909-026-09799-z)
Supplement: Supplementary file 3 — Supplementary Material 3. [file 12909_2026_9799_MOESM3_ESM.docx]

**Supplementary 5**

| **Domain / analysis** | **N** | **Descriptive pattern** | **Test results** | **Effect size** |
| --- | --- | --- | --- | --- |
| Obstetrics: baseline standard device (T0a) vs immediate post-training POCUS (T0b) | 22 | 4.03 to 3.85; mean difference = -0.18 | paired t-test: p = .591 | dz = -0.12 |
| Obstetrics: longitudinal omnibus test (T0b-T3) | 19 | 4.06, 4.92, 4.95, 5.11 | mixed-effects model: p < .001; Friedman: p < .001 | dz T0b to T3 = 1.02 [0.66, 1.62] |
| Obstetrics: pairwise T0b vs T1 | 19 | +0.85 | t-test: p = .002; Bonferroni p = .014 | dz = 0.82 |
| Obstetrics: pairwise T0b vs T2 | 19 | +0.89 | t-test: p = .005; Bonferroni p = .027 | dz = 0.74 |
| Obstetrics: pairwise T0b vs T3 | 19 | +1.05 | t-test: p < .001; Bonferroni p = .002 | dz = 1.02 |
| Gynecology: baseline standard device (T0a) vs immediate post-training POCUS (T0b) | 22 | 4.89 to 4.57; mean difference = -0.32 | paired t-test: p = .257 | dz = -0.25 |
| Gynecology: longitudinal omnibus test (T0b-T3) | 19 | 4.66, 5.57, 5.52, 5.84 | mixed-effects model: p < .001; Friedman: p = .007 | dz T0b to T3 = 0.90 [0.51, 1.49] |
| Gynecology: pairwise T0b vs T1 | 19 | +0.91 | t-test: p = .002; Bonferroni p = .015 | dz = 0.81 |
| Gynecology: pairwise T0b vs T2 | 19 | +0.86 | t-test: p = .013; Bonferroni p = .077 | dz = 0.63 |
| Gynecology: pairwise T0b vs T3 | 19 | +1.18 | t-test: p < .001; Bonferroni p = .006 | dz = 0.90 |
