# Supplementary material for: Physicians’ attitudes and perceived diagnostic confidence in point-of-care ultrasound in gynecology and obstetrics (GO-POCUS): a prospective single-center implementation study with structured training
Source: BMC Med Educ. 2026 Jun 29;26:1043. doi: 10.1186/s12909-026-09799-z (PMC13321536; doi:10.1186/s12909-026-09799-z)
Supplement: Supplementary file 4 — Supplementary Material 4. [file 12909_2026_9799_MOESM4_ESM.docx]

**Supplementary 4**

**Cluster-based analysis of obstetric diagnostic confidence**

**A. Cluster descriptives**

| **Cluster** | **Timepoint** | **N** | **Mean** | **SD** |
| --- | --- | --- | --- | --- |
| **Focused bedside / rapid assessment** | **T0b** | **19** | **4.83** | **1.24** |
| **Focused bedside / rapid assessment** | **T1** | **19** | **5.79** | **1.04** |
| **Focused bedside / rapid assessment** | **T2** | **19** | **5.89** | **1.05** |
| **Focused bedside / rapid assessment** | **T3** | **19** | **6.14** | **0.93** |
| **Advanced obstetric assessment** | **T0b** | **19** | **3.45** | **1.03** |
| **Advanced obstetric assessment** | **T1** | **19** | **4.22** | **1.56** |
| **Advanced obstetric assessment** | **T2** | **19** | **4.20** | **1.40** |
| **Advanced obstetric assessment** | **T3** | **19** | **4.28** | **1.39** |

**B. Cluster contrasts**

| **Contrast** | **N** | **Mean difference** | **95% CI** | **t-test p** | **Wilcoxon p** | **Cohen’s dz** |
| --- | --- | --- | --- | --- | --- | --- |
| **T0b: focused bedside vs. advanced obstetrics** | **19** | **1.38** | **[0.79, 1.96]** | **< .001** | **< .001** | **1.13** |
| **T1: focused bedside vs. advanced obstetrics** | **19** | **1.57** | **[0.92, 2.22]** | **< .001** | **< .001** | **1.16** |
| **T2: focused bedside vs. advanced obstetrics** | **19** | **1.69** | **[1.08, 2.31]** | **< .001** | **< .001** | **1.32** |
| **T3: focused bedside vs. advanced obstetrics** | **19** | **1.86** | **[1.17, 2.56]** | **< .001** | **< .001** | **1.29** |
| **Focused bedside / rapid assessment: T0b vs. T3** | **19** | **1.32** | **[0.79, 1.84]** | **< .001** | **< .001** | **1.22** |
| **Advanced obstetric assessment: T0b vs. T3** | **19** | **0.83** | **[0.28, 1.39]** | **.005** | **.009** | **0.72** |

**Note. Focused bedside / rapid-assessment scenarios included fetal bradycardia, fetal vitality assessment in the second/third trimester, placental localization, and amniotic fluid assessment. Advanced obstetric assessment scenarios included fetal biometry, cervical length assessment, fetal growth abnormalities, umbilical artery Doppler, and fetal growth restriction. Mean differences for same-timepoint contrasts represent focused bedside minus advanced obstetric assessment. Mean differences for within-cluster contrasts represent T3 minus T0b.**
