# Supplementary material for: Physicians’ attitudes and perceived diagnostic confidence in point-of-care ultrasound in gynecology and obstetrics (GO-POCUS): a prospective single-center implementation study with structured training
Source: BMC Med Educ. 2026 Jun 29;26:1043. doi: 10.1186/s12909-026-09799-z (PMC13321536; doi:10.1186/s12909-026-09799-z)
Supplement: Supplementary file 5 — Supplementary Material 5. [file 12909_2026_9799_MOESM5_ESM.docx]

**Supplementary 1**

**STROBE Statement—Checklist of items that should be included in reports of *cohort studies***

|  | Item No | Recommendation |
| --- | --- | --- |
| **Title and abstract** | 1 | (*a*) Indicate the study’s design with a commonly used term in the title or the abstract  Title and Abstract: The title/abstract identify the study as a prospective single-centre, longitudinal, observational implementation study. |
|  |  | (*b*) Provide in the abstract an informative and balanced summary of what was done and what was found  Abstract: The abstract summarizes the study setting, participants, training, timepoints, outcomes, analyses, and main findings. |
| Introduction | | |
| Background/rationale | 2 | Explain the scientific background and rationale for the investigation being reported  Introduction/Background: paragraphs describe POCUS in obstetrics and gynecology, implementation requirements, and the limited real-world evidence. |
| Objectives | 3 | State specific objectives, including any prespecified hypotheses  End of Introduction/Background: The manuscript states the objective to investigate physicians' attitudes, perceived diagnostic confidence, and preference during POCUS implementation. |
| Methods | | |
| Study design | 4 | Present key elements of study design early in the paper  Methods, Study design: The paper describes a prospective, longitudinal, observational implementation study at the Department of Gynecology and Obstetrics, University Hospital Marburg. |
| Setting | 5 | Describe the setting, locations, and relevant dates, including periods of recruitment, exposure, follow-up, and data collection  Methods, Study design and Measurements: Setting: University Hospital Marburg, Germany. Recruitment dates were corrected to August 26, 2025 to January 28, 2026. Follow-up assessments were performed after 2 weeks, 1 month, and 3 months. Data collection: facilitated via Unipark platform |
| Participants | 6 | (*a*) Give the eligibility criteria, and the sources and methods of selection of participants. Describe methods of follow-up  Methods, Study design and Measurements: Eligible participants were physicians in postgraduate training and board-certified specialists in the department who participated voluntarily. Follow-up timepoints were T1, T2, and T3, facilitated via the Unipark platform. |
|  |  | (*b*) For matched studies, give matching criteria and number of exposed and unexposed  Not applicable. This was not a matched cohort study and did not include exposed or unexposed comparison groups. |
| Variables | 7 | Clearly define all outcomes, exposures, predictors, potential confounders, and effect modifiers. Give diagnostic criteria, if applicable  Methods, Measurements: The primary outcome was attitude, the secondary outcome perceived diagnostic confidence and ultrasound device preference. Clinical scenarios and questionnaire items are provided in the Supplementary Material. |
| Data sources/ measurement | 8* | For each variable of interest, give sources of data and details of methods of assessment (measurement). Describe comparability of assessment methods if there is more than one group  **Methods, Measurements:** Data sources and measurement procedures are described in the Methods section. Outcomes were assessed using repeated quantitative surveys with 7-point Likert scales for attitude, perceived diagnostic confidence, and device preference, supplemented by a dichotomous device-preference item. The same survey constructs were applied across timepoints where applicable, ensuring comparability of repeated measurements. |
| Bias | 9 | Describe any efforts to address potential sources of bias  Methods/Statistical analyses and Limitations: Non-parametric sensitivity analyses were used. The revised Limitations section explicitly acknowledges investigator bias, Hawthorne effects, demand characteristics, and missing exposure data |
| Study size | 10 | Explain how the study size was arrived at  Methods/Participants and Results/Participants: The study included participating physicians from a single department during early implementation. A formal sample size calculation was not performed, this is an exploratory implementation study. Study size resulted largely from the department´s size and availability of physicians. |
| Quantitative variables | 11 | Explain how quantitative variables were handled in the analyses. If applicable, describe which groupings were chosen and why  Methods, Statistical analyses: Likert items were averaged into composite scores, descriptive statistics, paired tests, mixed-effects models, Friedman tests, and corrected pairwise tests were used. Exploratory clustering is reported in Supplementary Material. |
| Statistical methods | 12 | (*a*) Describe all statistical methods, including those used to control for confounding  Methods, Statistical analyses: The manuscript reports descriptive analyses, paired t-tests, Wilcoxon tests, mixed-effects models, Friedman tests, Cochran's Q/McNemar tests, effect sizes, bootstrap CIs, and software. |
|  |  | (*b*) Describe any methods used to examine subgroups and interactions  Supplementary Material: An exploratory cluster-based analysis of obstetric diagnostic confidence was added as Supplementary, no interaction analyses were performed. |
|  |  | (*c*) Explain how missing data were addressed  Methods/Statistical analyses and Results/Participants: Longitudinal analyses were restricted to participants with complete POCUS assessments, response numbers and complete-case sample size are reported. |
|  |  | (*d*) If applicable, explain how loss to follow-up was addressed  Results/Participants: One participant was lost to follow-up at T3, longitudinal complete-case analyses included 19 participants with complete data from T0b through T3. |
|  |  | (*e*) Describe any sensitivity analyses  Methods, Statistical analyses. Wilcoxon signed-rank tests and Friedman tests were used as non-parametric sensitivity analyses. |
| Results | | |
| Participants | 13* | (a) Report numbers of individuals at each stage of study—eg numbers potentially eligible, examined for eligibility, confirmed eligible, included in the study, completing follow-up, and analysed  Results/Participants: A total of 22 physicians completed T0a and T0b, 21 responses were available at each follow-up; 19 participants had complete data for longitudinal analyses. |
|  |  | (b) Give reasons for non-participation at each stage  Results/Participants: Loss to follow-up is reported. Specific reasons for non-participation were not available. |
|  |  | (c) Consider use of a flow diagram  Figure 1 provides an overview of measurement timepoints and the structured educational program. No separate participant flow diagram was used. |
| Descriptive data | 14* | (a) Give characteristics of study participants (eg demographic, clinical, social) and information on exposures and potential confounders  Results/Participants and Table 1: Demographics include age, professional experience, sex, training level, and subspecialty focus. |
|  |  | (b) Indicate number of participants with missing data for each variable of interest  Results/Participants and Tables: The manuscript reports n values by variable and timepoint, all tables report missing data |
|  |  | (c) Summarise follow-up time (eg, average and total amount)  Methods/Measurements and Results: Follow-up assessments took place after 2 weeks, 1 month, and 3 months of independent POCUS use. |
| Outcome data | 15* | Report numbers of outcome events or summary measures over time  Results, Tables 2-6 and Figures 2-4: Summary measures over time are reported for attitude, diagnostic confidence, and device preference. |
| Main results | 16 | (*a*) Give unadjusted estimates and, if applicable, confounder-adjusted estimates and their precision (eg, 95% confidence interval). Make clear which confounders were adjusted for and why they were included  Results, Sections 3.3-3.5 and Supplementary Material: Mean differences, p values, effect sizes, and bootstrap 95% confidence intervals are reported where applicable. No confounder-adjusted models were performed. |
|  |  | (*b*) Report category boundaries when continuous variables were categorized  Not applicable. Continuous variables were not categorized for the main analyses. Likert scales ranged from 1 to 7. |
|  |  | (*c*) If relevant, consider translating estimates of relative risk into absolute risk for a meaningful time period  Not applicable. The study did not estimate relative risks or clinical event risks. |
| Other analyses | 17 | Report other analyses done—eg analyses of subgroups and interactions, and sensitivity analyses  Results and Supplementary Material: Non-parametric sensitivity analyses, post hoc comparisons, internal consistency analyses, and exploratory cluster-based analyses are reported. |
| Discussion | | |
| Key results | 18 | Summarise key results with reference to study objectives  Discussion, Principal findings and Further findings: Key findings are summarized for attitude, perceived diagnostic confidence, and device preference. |
| Limitations | 19 | Discuss limitations of the study, taking into account sources of potential bias or imprecision. Discuss both direction and magnitude of any potential bias  Discussion, Limitations: The sections discuss perceived confidence versus actual performance, lack of time-related outcomes, absence of later standard-device reassessment, small single-centre sample, T0b construct validity, missing POCUS-use logs, and potential biases. |
| Interpretation | 20 | Give a cautious overall interpretation of results considering objectives, limitations, multiplicity of analyses, results from similar studies, and other relevant evidence  Discussion and Conclusions: The manuscript uses cautious wording and interprets results as observed during early implementation accompanied by structured training, not as proof of a causal training effect. |
| Generalisability | 21 | Discuss the generalisability (external validity) of the study results  Discussion, Limitations and Future Research: Generalisability is discussed in relation to the single tertiary-care center, small sample size, and structured support context; larger multicenter studies are recommended. |
| Other information | | |
| Funding | 22 | Give the source of funding and the role of the funders for the present study and, if applicable, for the original study on which the present article is based  Statements and Declarations/Funding and Competing Interests: The manuscript states that no funds, grants, or other support were received for manuscript preparation, describes the SUCCESS program support, Open Access funding, and device provision by Butterfly Network with no role in study design, conduct, analysis, or interpretation. |

*Give information separately for exposed and unexposed groups.
